# Diesel Exhaust Activates and Primes Microglia: Air Pollution, Neuroinflammation, and Regulation of Dopaminergic Neurotoxicity

**DOI:** 10.1289/ehp.1002986

**Published:** 2011-05-11

**Authors:** Shannon Levesque, Thomas Taetzsch, Melinda E. Lull, Urmila Kodavanti, Krisztian Stadler, Alison Wagner, Jo Anne Johnson, Laura Duke, Prasada Kodavanti, Michael J. Surace, Michelle L. Block

**Affiliations:** 1 Department of Anatomy and Neurobiology, Virginia Commonwealth University Medical Campus, Richmond, Virginia, USA; 2 Environmental Public Health Division, National Health and Environmental Effects Research Laboratory, U.S. Environmental Protection Agency, Research Triangle Park, North Carolina, USA; 3 Oxidative Stress and Disease Laboratory, Pennington Biomedical Research Center, Louisiana State University System, Baton Rouge, Louisiana, USA; 4 Cellular and Molecular Pathology Branch, National Institute of Environmental Health Sciences, National Institutes of Health, Department of Health and Human Services, Research Triangle Park, North Carolina, USA; 5 Neurotoxicology Branch, Toxicity Assessment Division, National Health and Environmental Effects Research Laboratory, Office of Research and Development, U.S. Environmental Protection Agency, Research Triangle Park, North Carolina, USA

**Keywords:** air pollution, brain, microglia, neuroinflammation, oxidative stress, Parkinson’s disease

## Abstract

Background: Air pollution is linked to central nervous system disease, but the mechanisms responsible are poorly understood.

Objectives: Here, we sought to address the brain-region–specific effects of diesel exhaust (DE) and key cellular mechanisms underlying DE-induced microglia activation, neuroinflammation, and dopaminergic (DA) neurotoxicity.

Methods: Rats were exposed to DE (2.0, 0.5, and 0 mg/m^3^) by inhalation over 4 weeks or as a single intratracheal administration of DE particles (DEP; 20 mg/kg). Primary neuron–glia cultures and the HAPI (highly aggressively proliferating immortalized) microglial cell line were used to explore cellular mechanisms.

Results: Rats exposed to DE by inhalation demonstrated elevated levels of whole-brain IL-6 (interleukin-6) protein, nitrated proteins, and IBA-1 (ionized calcium-binding adaptor molecule 1) protein (microglial marker), indicating generalized neuroinflammation. Analysis by brain region revealed that DE increased TNFα (tumor necrosis factor-α), IL-1β, IL-6, MIP-1α (macrophage inflammatory protein-1α) RAGE (receptor for advanced glycation end products), fractalkine, and the IBA-1 microglial marker in most regions tested, with the midbrain showing the greatest DE response. Intratracheal administration of DEP increased microglial IBA-1 staining in the substantia nigra and elevated both serum and whole-brain TNFα at 6 hr posttreatment. Although DEP alone failed to cause the production of cytokines and chemokines, DEP (5 μg/mL) pretreatment followed by lipopolysaccharide (2.5 ng/mL) *in vitro* synergistically amplified nitric oxide production, TNFα release, and DA neurotoxicity. Pretreatment with fractalkine (50 pg/mL) *in vitro* ameliorated DEP (50 μg/mL)-induced microglial hydrogen peroxide production and DA neurotoxicity.

Conclusions: Together, these findings reveal complex, interacting mechanisms responsible for how air pollution may cause neuroinflammation and DA neurotoxicity.

Air pollution is a prevalent source of environmentally induced inflammation/oxidative stress, and each year millions of people are exposed to levels of air pollution above promulgated safety standards (Block and Calderón-Garcidueñas 2009). Importantly, air pollution has been strongly associated with deleterious central nervous system (CNS) effects, including increased stroke incidence (Chen 2010), decreased cognitive function (Calderón-Garcidueñas et al. 2008a), and Alzheimer’s disease (AD)-like or Parkinson’s disease (PD)-like neuropathology (Block and Calderón-Garcidueñas 2009). Although prospective epidemiology studies of PD and air pollution are unavailable at this time, elevated levels of manganese in the air have been associated with enhanced PD risk (Finkelstein and Jerrett 2007). Consistent with findings from human populations, animal studies in dogs, mice, and rats show that air pollution components cause neuroinflammation, oxidative stress, and DNA damage and up-regulate markers of neurodegenerative disease (Block and Calderón-Garcidueñas 2009). However, although evidence supports an effect of air pollution on CNS pathology and disease, underlying mechanisms of such effects are unknown (Block and Calderón-Garcidueñas 2009).

Diesel exhaust (DE) is a major constituent  of near-road and urban air pollution and is commonly used as a surrogate model of air pollution in health effects studies (Hesterberg et al. 2010; Ma and Ma 2002). CNS responses to DE have been documented: Exposure has been shown to affect electroencephalogram parameters in human subjects (Cruts et al. 2008). Animal studies in rats using a month-long inhalation model (Gerlofs-Nijland et al. 2010) and a model using 2-hr-long exposure by nose-only inhalation (van Berlo et al. 2010) have demonstrated that DE elevates proinflammatory factors in select brain regions. Recent studies have also shown gene expression changes in the rat cerebrum after perinatal exposure to DE (Tsukue et al. 2009). In addition, *in utero* exposure to DE has been shown to affect dopamine (DA) neurochemistry and cause motor deficits in mice (Suzuki et al. 2010; Yokota et al. 2009). Our previous *in vitro* work has shown that microglia are activated by DE particles (DEP) to produce extracellular superoxide through NADPH oxidase, which is selectively toxic to DA neurons (Block et al. 2004). Further exploring cellular mechanisms of DE’s CNS effects, we have also shown that DEP impair the blood–brain barrier and cause capillaries to release tumor necrosis factor-α (TNFα) *in vitro*, contributing to inflammation (Hartz et al. 2008). However, although DE causes neuroinflammation, perturbs DA neurochemistry, and impairs motor behavior, the cellular and molecular mechanisms driving these effects are poorly understood. In the present study, we used DE as a model of air pollution to further define the deleterious CNS effects and to begin to address the complex mechanisms that mediate pathology.

## Materials and Methods

*Reagents.* Standard reference material (SRM) 2975 Diesel Particulate Matter (industrial fork lift) was purchased from the National Institute for Standards and Technology (Gaithersburg, MD). We purchased lipopolysaccharide (LPS; strain O111:B4) from EMD Chemicals (Gibbstown, NJ); recombinant rat fractalkine from Leinco Technologies, Inc. (St. Louis, MO); and cell culture reagents from Invitrogen (Carlsbad, CA). [^3^H]Dopamine (DA; 28 Ci/mmol) was purchased from NEN Life Science (Boston, MA). We purchased the tyrosine hydroxylase (TH) antibody from Millipore (Billerica, MA); the ionized calcium-binding adaptor molecule 1 (IBA-1) antibody from Wako (Richmond, VA); the α-synuclein antibody from Millipore; and the biotinylated horse anti-mouse and goat anti-rabbit secondary antibodies from Vector Laboratories (Burlingame, CA). All other reagents were procured from Sigma-Aldrich Chemical Co. (St. Louis, MO).

*Animals.* For the *in vivo* studies, 12-week-old male Sprague-Dawley rats and 12- to 14-week-old male Wistar Kyoto (WKY) rats were purchased from Charles River Laboratories (Raleigh, NC). Animals were acclimated to the housing facility for 1 week before studies began. For the primary cell culture studies, timed-pregnant (gestational day 14) adult female Fisher 344 rats were purchased from Charles River Laboratories. Housing, breeding, and experimental use of the animals were performed in strict accordance with National Institutes of Heath guidelines. All animals were treated humanely and with regard for alleviation of suffering.

*Animal treatment.* Inhalation. DE was generated by a 30-kW (40 hp) four- cylinder indirect injection Duetz diesel engine (BF4M1008), as previously described (Gottipolu et al. 2009; Stevens et al. 2008), and animals were exposed to DE in exposure chambers [for details, see Supplemental Material, p. 3 (doi:10.1289/ehp.1002986)]. Rats were exposed 4 hr/day, 5 days/week, for 1 month to air or DE at concentrations of 0, 0.5, or 2 mg/m^3^, which are higher than typically encountered in ambient air but may be achieved during heavy traffic or occupational situations. This is an established model commonly used to explore the effects of air pollution (Gottipolu et al. 2009; Stevens et al. 2008).

Intratracheal (IT) DEP administration. Male Sprague-Dawley rats received either phosphate-buffered saline, pH 7.4 (control) or DEP (SRM 2975; 20 mg/kg) suspended in saline, as previously described (Arimoto et al. 2005) [for details, see Supplemental Material, pp. 3–4 (doi:10.1289/ehp.1002986)]. Although DEP are administered in a single bolus at a concentration (20 mg/kg) that is higher than typical environmental exposures, this well-defined, established model (Gottipolu et al. 2009) provides data on the possible effects of particulate exposures via the lungs, as opposed to exposures through nasal entry to the brain.

*DEP preparation for* in vitro *studies.* Nanometer-sized DEP were used as a model of ultrafine particulate matter (PM) and were prepared as described previously (Block et al. 2004) [for details, see Supplemental Material, p. 4 (doi:10.1289/ehp.1002986)]. The precise amount of PM reaching the brain is currently unknown. However, studies have demonstrated that 0.01–0.001% of inhaled nanometer-sized iridium and carbon particulate remain in the brain 24 hr after exposure (Kreyling et al. 2009). Based on the *in vivo* models used in the present study (DEP; 0.5 mg/m^3^, 2 mg/m^3^, and 20 mg/kg), the *in vitro* concentrations of nanometer-sized particles (5–50 μg/mL) fall within the current estimates of what may reach the brain.

*Mesencephalic neuron–glia cultures.* Rat ventral mesencephalic neuron–glia cultures were prepared using a previously described protocol (Liu et al. 2001) [for details, see Supplemental Material, p. 4 (doi:10.1289/ehp.1002986)].

*Cell lines.* The rat microglia HAPI (highly aggressively proliferating immortalized) cells were a generous gift from J.R. Connor (Cheepsunthorn et al. 2001) and were maintained at 37°C in Dulbecco’s modified Eagle’s medium supplemented with 10% fetal bovine serum, 50 U/mL penicillin, and 50 μg/mL streptomycin in a humidified incubator with 5% CO_2_/95% air.

*DA uptake assay.* We measured the ability of DA neurons to uptake [^3^H]DA using a previously reported method (Block et al. 2004) [for details, see Supplemental Material, pp. 4–5 (doi:10.1289/ehp.1002986)].

*Immunostaining* in vitro. Microglia were stained with the polyclonal antibody raised against IBA-1 protein, and DA neurons were detected with the polyclonal antibody against TH, as reported previously (Block et al. 2004) [see Supplemental Material, p. 5 (doi:10.1289/ehp.1002986)].

*Immunostaining* in vivo. Brains from rats treated with saline or DEP via IT were fixed in 4% paraformaldehyde and processed for immunostaining as described previously (Qin et al. 2004) [for details, see Supplemental Material, p. 6 (doi:10.1289/ehp.1002986)].

*ELISAS (enzyme-linked immunosorbent assays).* We measured levels of TNFα, interleukin-6 (IL-6), macrophage inflammatory protein-1α (MIP-1α), IL-1β, fractalkine, and RAGE (receptor for advanced glycation end products) in cell culture supernatant and brain homogenate using commercially available ELISA kits (R&D Systems, Minneapolis, MN). Levels of protein nitration in brain homogenate, a common marker of oxidative stress, were also measured by ELISA, per manufacturer instructions (Millipore, Temecula, CA). For all ELISAs, brain regions were homogenized in Cytobuster lysis buffer (EMD Chemicals), and 100 μg total protein was assayed per well.

We developed an indirect ELISA to quantitate relative amounts of IBA-1 expression in brain homogenate [for details, see Supplemental Material, p. 6 (doi:10.1289/ehp.1002986)]. We measured the amount of DA present in midbrain tissue using snap-frozen, dissected midbrain tissue homogenized in 0.1 M HCl and 0.1 mM EDTA and a commercially available kit from Genway Biotech Inc. (San Diego, CA), following manufacturer instructions.

*Nitrite assay.* Nitrite levels present in media were measured with Griess reagent, as reported previously (Block et al. 2004) [for details, see Supplemental Material, p. 7 (doi:10.1289/ehp.1002986)].

*Hydrogen peroxide assay.* Levels of hydrogen peroxide (H_2_O_2_) production in cell culture were determined as previously described (Werner 2003), with slight modifications [for details, see Supplemental Material, p. 7 (doi:10.1289/ehp.1002986)].

*Immunoblotting.* We performed immunoblotting as reported previously (Qin et al. 2004) [for details, see Supplemental Material, pp. 7–8 (doi:10.1289/ehp.1002986)].

*Quantitative real-time reverse transcriptase polymerase chain reaction (RT-PCR).* We measured levels of *TNF*α and *MIP-1*α mRNA by RT-PCR. Total RNA was extracted from the mouse olfactory bulbs using the RNA Easy kit (Qiagen, Valencia, CA) as described previously (Qin et al. 2004) [for details, see Supplemental Material, p. 8 (doi:10.1289/ehp.1002986)].

*Statistical analysis.* Data are expressed as raw values, percentage of control, fold increase from control, or the difference from control, where control values were set to 100%, 1, or 0 accordingly. Data for the treatment groups are expressed as mean ± SE. We assessed statistical significance with a one- or two-way analysis of variance followed by Bonferroni’s post hoc analysis using SPSS software (SPSS Statistics 19; IBM, Armonk, New York). A value of *p* < 0.05 was considered statistically significant.

## Results

**Figure 1:**
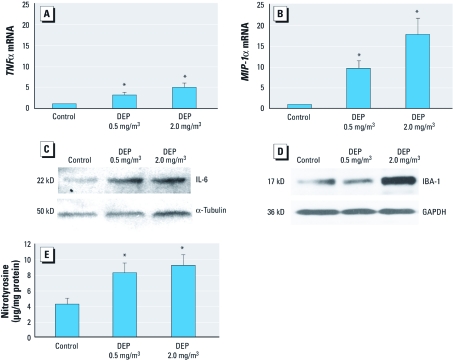
Inhalation exposure to DE elevates markers of neuroinflammation. WKY rats were exposed to 0 (air control), 0.5, or 2.0 mg/m^3^ DEP (*n* = 3/treatment group). *TNF*α (*A*) and *MIP‑1*α (*B*) mRNA levels (mean ± SE) were determined in olfactory bulbs using quantitative real-time RT-PCR; values represent the fold increase from control 2^–ΔΔCT^ normalized with α-tubulin and expressed as a percentage of control. Representative images show changes in IL‑6 (*C*) and IBA‑1 (*D*) in whole-brain homogenate assayed by Western blotting; GAPDH was used as a loading control. (*E*) Whole-brain homogenate was also assayed for nitrosylated protein by ELISA; values shown are mean ± SE. *p < 0.05, compared with controls.

*Inhalation exposure.* DE and neuroinflammation. To discern whether DE caused neuroinflammation at all, we measured proinflammatory mRNA expression in the olfactory bulb in rats exposed for 1 month by inhalation. DE caused a significant increase in both *TNF*α and *MIP-1*α mRNA expression in the olfactory bulbs (*p* < 0.05) (Figure 1A,B).

To explore whether neuroinflammation was generalized throughout the brain, we tested whole-brain homogenate from rats exposed for 1 month by inhalation. Data show that DE exposure increased expression of IL-6 (Figure 1C) and IBA-1 (microglial marker; Figure 1D), as measured by Western blot. DE also caused a significant elevation of protein nitration in whole-brain homogenate (Figure 1E). At the time of sacrifice, no cytokines were elevated in the serum in DE-exposed rats compared with controls (data not shown) (Gottipolu et al. 2009). These data suggest that DE exposure caused generalized neuroinflammation and oxidative stress that extended throughout the entire brain.

**Figure 2:**
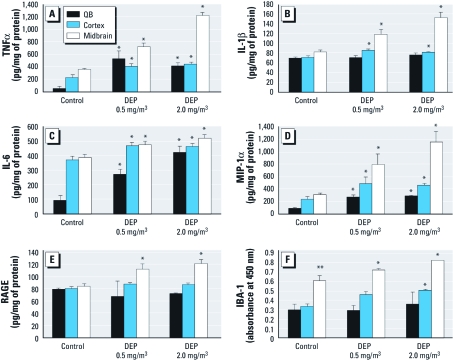
DE elevates microglial markers, cytokines, and chemokines in brain regions. WKY rats were exposed to 0 (air control), 0.5, or 2.0 mg/m^3^ DEP (*n* = 4/treatment group). Protein levels of TNFα (*A*), IL‑1β (*B*), IL‑6 (*C*), MIP‑1α (*D*), RAGE (*E*), and IBA‑1 (microglial marker; *F*) from the olfactory bulb (OB), cortex, and midbrain were measured by ELISA. In controls, IBA‑1 was significantly higher in the midbrain compared with other brain regions, indicating higher levels of microglia in the absence of exposure. Notably, the midbrain region also showed the highest levels of cytokines, chemokines, and microglial markers in response to DE. *p < 0.05, compared with the corresponding region in controls. **p < 0.05 for IBA‑1 in midbrain, compared with olfactory bulbs and cortex levels in controls.

DE and brain-region–specific neuroinflammation. We next addressed the effects of DE on neuroinflammation in brain regions known to be affected by neurodegenerative disease: the cortex (AD), midbrain (PD), and olfactory bulb (AD and PD) (Hawkes et al. 1997; Kovacs et al. 1998). Several cytokines were evaluated in tissue homogenates by ELISA. All three brain regions showed significant increases in TNFα protein expression in response to DE, with the greatest increase occurring in the midbrain (Figure 2A). The proinflammatory cytokine IL-1β was not increased in the olfactory bulbs but was significantly increased in the midbrain and cortex in response to both levels of DE exposure, with the most pronounced response in the midbrain (Figure 2B). All three brain regions showed increased expression of IL-6 protein in response to DE (Figure 2C) and increased levels of MIP-1α chemokine, with the highest MIP-1α levels expressed in the midbrain (Figure 2D).

In addition, we examined the effect of DE on levels of RAGE, which is elevated in the substantia nigra and frontal cortex in cases of early stages of parkinsonian neuropathology (Dalfo et al. 2005) and is key to how microglia identify many neurotoxic stimuli (Fang et al. 2010). Interestingly, DE elevated RAGE expression, but only in the midbrain (Figure 2E).

Notably, in controls, the midbrain showed significantly higher levels of the microglial marker IBA-1 compared with either the cortex or the olfactory bulb (Figure 2F), suggesting that numbers of microglia are higher in the midbrain under normal conditions. IBA-1 protein was significantly increased in the cortex after high-DE exposure (2.0 mg/m^3^) but was increased in the midbrain in response to both DE exposure levels (Figure 2F). Combined with evidence of higher levels of microglia and a more pronounced proinflammatory response in the midbrain, these findings support a greater vulnerability of the midbrain to air-pollution–induced neuroinflammation compared with other brain regions.

**Figure 3:**
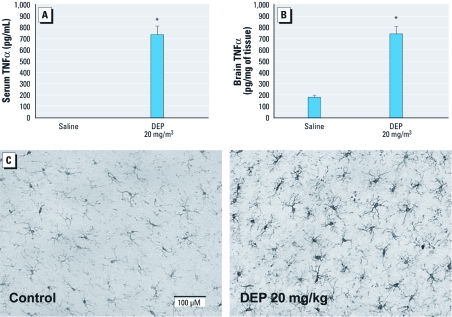
DEP cause TNFα production and microglia activation *in vivo*. Sprague-Dawley rats were treated IT with saline or 20 mg/kg DEP (*n* = 3/treatment group). Serum (*A*) and brain (*B*) TNFα levels were determined 6 hr postexposure, as measured by ELISA. (*C*) Representative images of immunohistochemically stained substantia nigra sections at 20 hr post-DEP treatment show enhanced microglial IBA‑1 expression, consistent with mild microglial activation. Bar = 100 μm. *p < 0.05, compared with controls.

*DEP and neuroinflammation and systemic inflammation* in vivo. Air pollution comprises numerous compounds, including gases and PM. Because the DE inhalation exposure contained particulate and gas-phase components (primarily carbon monoxide and nitric oxide; see Gottipolu et. al. 2009), it is unclear which components of DE are responsible for CNS effects. To test the ability of PM to induce neuroinflammation, we administered the particles (DEP; SRM 2975) to rats by IT installation and measured TNFα levels in serum and whole-brain homogenate by ELISA. DEP caused significant TNFα elevation in both the serum and the brain (Figure 3A,B). Immunostaining of IBA-1 showed that DE did not cause acute changes in microglial morphology at 20 hr posttreatment but did up-regulate IBA-1 expression (indicated by darker staining in Figure 3C), consistent with mild microglial activation. These results indicate that DEP can cause systemic TNFα elevation, increase brain TNFα, and activate microglia *in vivo*.

**Figure 4:**
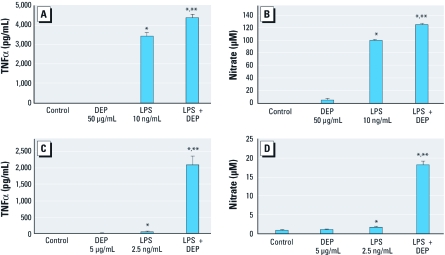
DEP amplify the microglial proinflammatory response to LPS *in vitro*. (*A* and *B*) HAPI microglia were pretreated for 30 min with DEP followed by LPS treatment; supernatant was collected for analysis at 3 hr for TNFα (*A*) and at 24 hr for nitrite (*B*). (*C* and *D*) Primary mesencephalic neuron–glia cultures were pretreated for 30 min with low concentrations of DEP (5 μg/mL) followed by low concentrations of LPS (2.5 ng/mL); supernatants were collected for analysis at 3 hr for TNFα (*C*) and at 24 hr for nitrite (*D*). *p < 0.05, compared with controls (*n* = 3). **p < 0.05, compared with LPS alone (*n* = 3).

*DEP and microglia.* It is likely that nanosized DEP particulates and leachable components translocate to the systemic circulation through the pulmonary capillary bed (Valavanidis et al. 2008). Recent studies have shown that PM (Wang et al. 2008) from air pollution (Calderón-Garcidueñas et al. 2008b) actually reaches the brain, which may be one mechanism by which CNS effects occur. Using an *in vitro* model, we also tested the ability of DEP to modulate ongoing neuroinflammation. Microglia cultures pretreated with DEP had significantly enhanced levels of TNFα (Figure 4A) and nitrite (Figure 4B) in response to LPS. We also observed this DEP priming response in primary neuron–glia cultures, where a low, nonneurotoxic concentration of DEP (5 μg/mL) amplified LPS-induced TNFα (Figure 4C) and nitrite (Figure 4D). Together, these data indicate that DEP prime microglia, increasing their sensitivity to additional proinflammatory stimuli. Additional proinflammatory stimuli could include ongoing neurodegeneration or perhaps the peripheral cytokine response as it transfers to the brain.

*DE and DA neurotoxicity* in vivo *and* in vitro. We have previously shown *in vitro* that DEP are selectively toxic to DA neurons through microglial NADPH oxidase activation and the consequent production of extracellular superoxide (Block et al. 2004). Interestingly, we did not observe evidence of DA neurotoxicity 24 hr after IT DEP exposure based on immunohistochemistry and staining of TH neurons (data not shown). Brain tissue sections were not available for the DE inhalation study, but we did not observe significant differences in the DA content in the midbrain region based on DA ELISA and α-synuclein content (Western blot) after DE exposure (data not shown).

**Figure 5:**
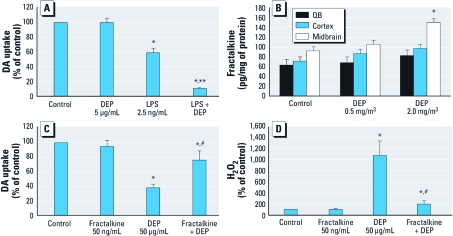
Effect of fractalkine on DEP-induced DA neurotoxicity *in vitro* and *in vivo*. DEP synergistically enhanced inflammation-mediated loss of DA neuron function. (*A*) DA neuron function in neuron–glia cultures 7–9 days after DEP exposure was measured with the [^3^H]DA uptake assay. (*B*) DE inhalation increased fractalkine levels in olfactory bulb (OB), cortex, and midbrain of rats; WKY rats were exposed to 0 (air control), 0.5, or 2.0 mg/m^3^ DEP (*n* = 4/treatment group) for 1 month, and fractalkine levels were measured by ELISA. (*C* and *D*) Primary mesencephalic neuron–glia cultures were pretreated for 30 min with fractalkine (100 pg/mL) followed by DEP (50 μg/mL) exposure (*n* = 3/treatment group). (*C*) DEP-induced loss of DA neuron function was reduced by fractalkine 7 days after treatment as measured by DA uptake assay. (*D*) DEP-induced H_2_O_2_ in microglia was reduced by fractalkine 3 hr after treatment. *p < 0.05, compared with controls. **p < 0.05, compared with LPS alone. ^#^*p* < 0.05, compared with DEP alone.

We also investigated whether the low nonneurotoxic concentration of DEP (5 μg/mL) that enhanced the microglial proinflammatory response to LPS (Figure 4C,D)affected DA neurotoxicity in response to LPS. Pretreatment with DEP for 30 min significantly enhanced LPS-induced loss of DA neuron function, as measured by DA uptake (Figure 5A). This suggests that exposure to low levels of air pollution that fail to initiate neurotoxicity alone may instead enhance additional proinflammatory triggers or ongoing neurodegenerative processes.

In an effort to understand why DA neuron damage was absent in the DE *in vivo* models, we began to explore the effect of DE on compensatory mechanisms in the brain that could counteract the neurotoxic effects of neuroinflammation. Fractalkine is a chemokine expressed by neurons, and the receptors are exclusively on microglia (Cardona et al. 2006). Solublized fractalkine is a key regulator of the microglial proinflammatory response, where it is has been shown to protect against microglia-mediated DA neurotoxicity *in vitro* and in PD models *in vivo* (Re and Przedborski 2006). Here, we show that fractalkine was elevated only in the midbrain region after 1 month of DE exposure (2.0 mg/m^3^) via inhalation (Figure 5B). *In vitro* studies revealed that soluble fractalkine attenuated DE-induced microglia H_2_O_2_ production (Figure 5D) and DEP-induced loss of DA neuron function *in vitro* (Figure 5C). Although elevated fractalkine expression did not abolish DE-induced neuroinflammation *in vivo*, fractalkine may attenuate the proinflammatory response to nonneurotoxic levels.

## Discussion

There is increasing evidence that environmental inhalation exposures may result in neuroinflammation and DA neuropathology (Antonini et al. 2009; Choi et al. 2010; Sriram et al. 2010; Verina et al. 2011), but the mechanisms are poorly understood. The present study employed *in vivo* and *in vitro* DE models to explore the mechanisms through which air pollution causes neuroinflammation and microglial activation, as well as the relevance of DE exposure for DA neuron survival. Here, we show that DE caused oxidative stress (i.e., protein nitration) and activated microglia *in vivo* (Figures 1–3). After 1 month of inhalation exposure, the IBA-1 microglial marker was up-regulated, particularly in the midbrain region, which contains the substantia nigra (Figure 2). At 24 hr after IT DEP administration, immunohistochemical analysis showed that DEP up-regulated IBA-1 on microglial cells, without obvious differences in morphology or cell number (Figure 3), a response similar to microglial activation previously observed in response to systemic LPS administration (Qin et al. 2007). Because immunohistochemical analysis of the month-long DE exposure was unavailable, we could not determine whether IBA-1 levels increased because of increased microglial numbers or because of up-regulated IBA-1 protein in the microglial membranes. However, DEP triggered H_2_O_2_ production from microglia *in vitro* (Figure 5). Further studies are needed to determine whether air pollution causes increased monocyte trafficking to the brain, qualitative changes in recruited cell populations (circulating monocytes vs. bone marrow), or proliferation of parenchymal microglia in vulnerable brain regions.

Our present work provides evidence of generalized proinflammatory cytokine elevation throughout the brain (Figure 1), with the greatest proinflammatory response to DE observed in the midbrain (Figure 2). This distinction is important because, consistent with previous reports (Kim et al. 2000), the midbrain also expressed the highest levels of microglial markers at rest and the greatest elevation of these markers in response to DE, which suggests that microglia may mediate a regional vulnerability to the neuroinflammatory effects of air pollution (Figure 2). These data also suggest that DE-induced neuroinflammation may be due in large part to a systemic response that affects the entire brain, rather than a local effect mediated solely by direct exposure through the olfactory bulb, a favored pathway of PM entry into the brain (Oberdörster et al. 2004). In fact, although the olfactory bulb showed elevation of some proinflammatory factors with DE exposure, it also failed to show up-regulation of IBA-1, RAGE, fractalkine, or IL-1β in response to DE, indicating a less pronounced proinflammatory response in this region (Figure 2). Notably, IT administration of DEP also resulted in increased TNFα production in whole-brain homogenates and activated microglia morphology in the substantia nigra (Figure 3), further supporting the hypothesis that nasal entry through the olfactory bulb may not be necessary for DE to cause neuroinflammation.

There is increasing evidence that systemic inflammation may contribute to neurodegenerative diseases (Perry et al. 2010). We (Qin et al. 2007) and others (Ling et al. 2002, 2006, 2009; Wang et al. 2009) have previously shown that systemic LPS administration causes neuroinflammation that persists long after the peripheral proinflammatory response has resolved, resulting in delayed and progressive (Qin et al. 2007) DA neurotoxicity. Consistent with our prior studies using LPS in adult animals that showed pronounced neuroinflammation that persisted in absence of the initiating peripheral proinflammatory trigger (Qin et al. 2007), we failed to see peripheral cytokines after 1 month of DE inhalation exposure, despite evidence of elevated neuroinflammation. In contrast, we observed increases in both serum and brain TNFα (6 hr and 24 hr) after IT administration of a single large dose of DEP (Figure 3). However, differences in cytokine responses between the two models may be due to kinetics, differences in the concentration of DEP versus DE, and/or chemical differences between the two exposures (i.e., gaseous components such as carbon monoxide and nitrogen oxides). Microglia and astrocytes did not produce cytokines or chemokines in response to DEP *in vitro*, suggesting that although PM reaching the brain may cause microglia-derived oxidative stress, systemic effects may be necessary to produce a comprehensive neuroinflammatory response that includes proinflammatory factor production. Further, DEP enhanced microglial responses to proinflammatory effects of LPS (Figure 4), indicating that interaction between DEP and systemic cytokines may amplify neuroinflammation. Thus, DE exposure caused increased levels of systemic cytokines that may contribute to microglial activation and the proinflammatory milieu of the brain.

We have previously shown *in vitro* that DEP activate microglia and are selectively toxic to DA neurons through microglia-derived reactive oxygen species (Block et al. 2004). In the present study, DE activated microglia, elevated neurotoxic cytokines in the midbrain, and induced oxidative stress *in vivo* (Figures 1 and 2), but we found no evidence of DA neurotoxicity *in vivo*. However, the longest exposure was only 1 month, and our prior research indicated that both aging and chronic microglial activation are needed to culminate in DA neuron death *in vivo*, suggesting that longer exposures and/or aging may be necessary for DE-induced neuroinflammation to initiate neurodegeneration *in vivo*.

To discern why DE failed to cause DA neurotoxicity *in vivo*, we shifted our focus to homeostatic mechanisms designed to regulate microglia activation. Fractalkine is a chemokine produced by neurons that is cleaved to become a soluble antiinflammatory signal for microglia (Cardona et al. 2006). In fact, microglia are reported to be the only CNS cell type that expresses fractalkine receptors, and fractalkine-knockout mice have enhanced neuroinflammation and elevated DA neurotoxicity in response to 1-methyl-4-phenyl-1,2,3,6-tetrahydropyridine *in vivo* (Re and Przedborski 2006). In the present study, DE elevated fractalkine expression only in the midbrain *in vivo*. *In vitro*, fractalkine inhibited DE-induced H_2_O_2_ production from microglia and protected against DE-induced DA neurotoxicity in midbrain neuron–glia cultures. Recent reports indicated that fractalkine (Duan et al. 2008) and fractalkine receptors (Wynne et al. 2010) decrease in the aged brain, which further supports the premise that aging may be critical for air-pollution–induced neuroinflammation to cause neurotoxicity *in vivo*. In addition, lower and seemingly benign concentrations of DEP *in vitro* shifted microglia to a primed phenotype, resulting in a more pronounced proinflammatory response and amplified neurotoxicity with additional stimuli (Figure 5). Thus, even when not immediately toxic, air pollution may amplify ongoing neuroinflammation and associated neuron damage. Further studies are needed to discern the role of aging, fractalkine, and priming in the deleterious effects of air pollution.

## Conclusion

Here we show that DE caused microglial activation and up-regulation of oxidative stress, pattern recognition receptors, neurotoxic cytokines, and chemokines in the rat brain. The midbrain expressed the highest levels of the IBA-1 microglial marker in control animals and produced the greatest response to DE, suggesting regional vulnerability. DEP activated microglia *in vitro* without increasing cytokine or chemokine production, but IT administration of DEP elevated both serum and brain TNFα levels *in vivo*, suggesting a key role for systemic inflammation. Indeed, our findings suggest that DEP may interact with ongoing inflammation to amplify the proinflammatory response (i.e., priming), which may be neurotoxic. This may be particularly relevant for individuals with active systemic inflammation or neurodegenerative disease. Although we did not detect significant loss of DA neurons *in vivo* in the models tested here, results suggest that fractalkine, which was elevated after DE exposure *in vivo*, may be responsible for adaptation by inhibiting DE-induced DA neurotoxicity, at least temporarily. Together, these findings reveal complex, interacting mechanisms responsible for how air pollution may cause neuroinflammation and DA neurotoxicity [see Supplemental Material, Figure 1 (doi:10.1289/ehp.1002986)] and may be particularly relevant to the etiology of PD.

## Supplemental Material

(584 KB) PDF
