# Supplementary material for: Diesel Exhaust Activates and Primes Microglia: Air Pollution, Neuroinflammation, and Regulation of Dopaminergic Neurotoxicity
Source: Environ Health Perspect. 2011 May 11;119(8):1149–55. doi: 10.1289/ehp.1002986 (PMC3237351; doi:10.1289/ehp.1002986)
Supplement: (584 KB) PDF [file ehp.1002986.s001.pdf]

SUPPLEMENTAL MATERIAL

Diesel Exhaust Activates & Primes Microglia: Air Pollution, Neuroinflammation, & Regulation of Dopaminergic Neurotoxicity

[1]Shannon Levesque, [1]Thomas Taetzsch, [1]Melinda E. Lull, [2]Urmila Kodavanti, [3]Krisztian Stadler, [1]Alison Wagner, [4]Jo Anne Johnson, [1]Laura Duke, [5]Prasada Kodavanti, [1]Michael J. Surace & [1]Michelle L. Block.

[1]Department of Anatomy and Neurobiology, Virginia Commonwealth University Medical Campus, Richmond, VA 23298, USA, [2]Environmental Public Health Division, National Health and Environmental Effects Research Laboratory, U.S. Environmental Protection Agency, Research Triangle Park, North Carolina 27711. [3] Oxidative Stress and Disease Laboratory, Pennington Biomedical Research Center, Louisiana State University System, Baton Rouge, LA 70808. [4]Cellular and Molecular Pathology Branch, National Institute of Environmental Health Sciences, National Institutes of Health, Department of Health and Human Services, Research Triangle Park, North Carolina, USA. [5] Neurotoxicology Branch, Toxicity Assessment Division, B 105-06, National Health and Environmental Effects Research Laboratory, Office of Research and Development, US Environmental Protection Agency, Research Triangle Park, NC 27711, USA.

***Address correspondence to:** Michelle L. Block, Ph.D.
Department of Anatomy & Neurobiology
Sanger Hall, Room 9-048
1101 E. Marshall Street
Virginia Commonwealth University Medical Campus
Box 980709
Richmond, VA 23298-0709
Phone: (804) 827-1967 **Fax:** (804) 828-4977

TABLE OF CONTENTS

**I.**	**SUPPLEMENTAL METHODS**	
	A. Animal treatment- Inhalation	Page 3
	B. Animal treatment- Intratracheal (IT) DEP administration	Page 3
	C. DEP preparation for in vitro studies	Page 4
	E. Mesencephalic neuron-glia cultures	Page 4
	D. DA uptake assay	Page 4
	F. Immunostaining – in vitro	Page 5
	G. Immunostaining – in vivo	Page 6
	H. IBA-1 ELISA	Page 6
	I. Nitrite assay	Page 7
	J. Hydrogen peroxide assay	Page 7
	K. Immunoblotting	Page 7
	L. Quantitative Real-time PCR	Page 8
**II.**	**SUPPLEMENTAL FIGURES**	
	A. Figure S1. Diesel exhaust causes neuroinflammation through diverse pathways.	Page 9
**III.**	**SUPPLEMENTAL REFERENCES**	Page10

I. SUPPLEMENTAL METHODS

Animal treatment- Inhalation

Diesel exhaust was generated by a 30-kW (40hp) four-cylinder indirect injection Duetz diesel engine (BF4M1008), as previously described (Stevens et al. 2008; Gottipolu et al. 2009). Specifically, a small portion of the whole DE was passed through a two stage dilution system, before being routed to the Hinner exposure chambers. WYK rats were exposed to one of three levels of DE (0, 0.5, and 3.0 mg/m^3) in the Hinner chambers, where temperature (22°C) and relative humidity (55-60%) were constantly monitored and maintained. Exposures were conducted for 4 hrs/day, 5 days/week, for 4 consecutive weeks. The DE exposure contained both particulate matter and gas, where the characteristics of the DE were monitored throughout the exposure. Integrated 4 hr filter samples (14.11/min) were collected daily from each chamber and analyzed gravimetrically to determine particle concentrations. Organic/elemental carbon concentrations (OC/EC) ratio of the airborne particulate matter was determined to remain constant throughout the treatment at 3.0 ± 0.03 by a thermal/optical carbon analyzer (Sunset Laboratory Inc., model 107, Tigard, OR). Particle geometric mass median aerodynamic diameter was consistently determined to be below 225 nm in both the 0.05 and 2.0 mg/m^3 DEP treatments. With reference to the gaseous content of the exposure, the carbon monoxide, nitrogen oxide, nitrogen dioxide, and sulfur dioxide were monitored, where the results are published in Gottipolu et al. (Gottipolu et al. 2009). Animals were sacrificed with sodium pentobarbital (50-100 mg/kg).

Animal treatment- Intratracheal (IT) DEP administration

Male Sprague-Dawley rats received either phosphate-buffered saline at pH 7.4 (control) or DEP (20 mg/kg, SRM 2975) suspended in saline, as previously described (Arimoto et al. 2005). Both saline and DEP were administered by the intratracheal (IT) route under anesthesia with 4% isoflurane (Baxter, Deerfield, IL). The DEP suspension was sonicated for 15 minutes under cooling conditions. Animals were sacrificed with sodium pentobarbital (50-100 mg/kg) 6 and 20 hrs after treatment and blood and brain tissue was collected.

This model of DEP exposure in Sprague-Dawley rats has been previously shown to synergistically enhance lung free radical generation in response to LPS at 6 hrs post treatment (Arimoto et al. 2005).

DEP preparation for in vitro studies

Nanometer-sized DEP were used as a model of ultra-fine particulate matter and were prepared according to Block et al. (Block et al. 2004). Briefly, 2 mg of DEP (DEP, NIST, SRM 2975) were added to 40 ml of treatment media and vortexed (<20 s), followed by sonication for 15 min. The DEP suspension was then filtered through a 0.22 μm filter (Millipore, Billerica), immediately diluted to appropriate concentrations, and immediately added to the culture. The chemical and particle characteristics of the DEP sample are readily available at NIST (https://www-s.nist.gov/srmors/view_cert.cfm?srm=2975) (NIST 2000). The endotoxin level in DEP (SRM 2975) was quantified using a commercially available kit (GenScript, Piscataway, NJ) and negligible amounts were found: DEP = 0.098 EU.

Mesencephalic neuron-glia cultures

Rat ventral mesencephalic neuron-glia cultures were prepared using a previously described protocol (Liu et al. 2001). Briefly, midbrain tissues were dissected from day 14 Fisher 344 rat embryos. Cells were dissociated via gentle mechanical trituration in minimum essential medium (MEM) and immediately seeded ($5x10^5$/well) in poly D-lysine (20 μg/ml) pre-coated 24-well plates. Cells were seeded in maintenance media and exposed to the treatment media, as described previously (Liu et al. 2001). Three days after seeding, the cells were replenished with 500 μL of fresh maintenance media. Cultures were treated 7 days after seeding.

DA uptake assay

The ability of dopaminergic neurons to uptake [^{3}H] DA was measured, using previously reported method (Block et al. 2004). Cells were incubated in Krebs-Ringer buffer (16 mM NaH_2PO_4, 16 mM NaH_2PO_4, 1.2 mM $MgSO_4$, 1.3 mM EDTA, 4.7 nM KCL, 16 mM Na_2HPO_4) for 15 minutes at 37ºC with 1 μM [^{3}H] DA.

Non-specific uptake was blocked for DA with 10 μM mazindol. After incubation, cells were washed three times with 1 mL/well of ice-cold Krebs-ringer buffer. Cells were then lysed with 0.5 mL/well of 1 N NaOH and mixed with 15 mL of scintillation fluid. Radioactivity was measured on a scintillation counter, where specific [^{3}H] DA uptake was calculated by subtracting the mazindole counts from the wells without the uptake inhibitors.

Immunostaining – in vitro

Microglia were stained with the polyclonal antibody raised against IBA-1 protein (Shapiro et al. 2009) and dopaminergic neurons were detected with the polyclonal antibody against tyrosine hydroxylase (TH), as reported previously (Block et al. 2004). Briefly, cells were fixed for 20 min at room temperature in 3.7% formaldehyde diluted in phosphate buffered saline (PBS). After washing twice with PBS, the cultures were treated with 1% hydrogen peroxide for 10 min. The cultures were again washed three times with PBS and then incubated for 40 min with blocking solution (PBS containing 1% bovine serum albumin, 0.4% Triton X-100, and 4% appropriate serum: normal horse serum for NeuN and normal goat serum for TH, GFAP, or IBA-1 staining). The cultures were incubated overnight at 4°C with the primary antibody diluted in DAKO antibody diluent, and the cells were washed three times for 10 min each in PBS. Next, the cultures were incubated for 1 hr with PBS containing 0.3% Triton X-100 and the appropriate biotinylated secondary antibody (IBA-1 and TH: goat anti-rabbit antibody, 1:277). After washing three times with PBS, the cultures were incubated for 1 hr with the Vectastain ABC reagents and diluted in PBS containing 0.3% Triton X-100. Cells were then washed two times with PBS, the bound complex was visualized by incubating cultures with 3,3'-diaminobenzidine, and the urea-hydrogen peroxide tablets were dissolved in water. Color development was halted by removing the reagents and washing the cultures twice with fresh PBS. Images were visualized on an inverted microscope (Zeiss Axio Observer A1, Carl Zeiss MicroImaging Thornwood, NY) and captured with the Axio Cam MRc5 imaging system (Carl Zeiss MicroImaging Thornwood, NY).

Immunostaining- in vivo

Brains from rats treated with saline or DEP via IT were fixed in 4% paraformaldehyde and processed for immunostaining as described previously (Qin et al. 2004). Twenty-four consecutive brain slices (35 μm thickness), which encompassed the entire substantia nigra pars compacta, were collected. Eight evenly-spaced brain slices from saline or DEP-treated animals were immunostained with an antibody against TH or IBA-1. Dopaminergic neurons were detected with the polyclonal antibody against tyrosine hydroxylase (TH). Microglia were stained with rat monoclonal antibody raised against the rabbit anti-Iba1 antibody, respectively. Immunostaining was visualized by using 3,3'-diaminobenzidine and urea-hydrogen peroxide tablets from Sigma.

IBA-1 ELISA

We developed an indirect ELISA to quantitate relative amounts of IBA-1 expression in brain homogenate. Nunc-immuno^{TM} 96 well plates were coated with 100 μg (1 μg/μl) of brain homogenate per well (samples run in duplicates) at 4°C overnight and washed three times with 0.1% Tween 20 in PBS. The plate was then blocked for 2 h with 300 μl of the blocking buffer (1% bovine serum albumin and 5% sucrose in PBS). IBA-1 antibody (1:1000, Wako, Richmond, VA) was suspended in reagent diluent (1% bovine serum albumin in PBS) and 100 μl was added to each well for 2 hrs at room temperature. The plate was washed three times with 0.1% Tween 20 in PBS. Then, 100 μl (1 μg/ml) of anti-rabbit horseradish peroxidase-conjugated antibody (Vector Laboratories, Burlingame, CA) in reagent diluent was added for 1 h and washed three times with 0.1% Tween 20 in PBS. 3,3',5,5'-Tetramethylbenzidine solution was added (100 μl, Sigma Aldrich Chemical Company, St. Louis, MO) for 30 min. Finally, 50 μl of stop solution was added (1 N H_2SO_4) and the microplate was read at an absorbance of 450 nm. The ELISA measures the relative amount of IBA-1 in protein in tissue homogenate samples, thus results are reported as absorbance at 450 nm.

Nitrite assay

Nitrite levels present in media were measured using our previously reported method with Griess reagent (Block et al. 2004). As an indirect footprint/indicator of nitric oxide production, the amount of nitrite accumulated in culture supernatant was determined with a colorimetric assay using Griess reagent [1% sulfanilamide, 2.5% H_3PO_4, 0.1% N- (1-naphthyl) ethylenediamine dihydrochloride] (Green et al. 1982) as previously reported (Block et al. 2004). Briefly, equal amounts of Griess reagent and of culture supernatant were incubated in a dark at room temperature for 10 minutes. After incubation, the absorbance at 540 nm was determined with the PHERAstar microplate spectrophotometer. The sample nitrite concentration was determined from a sodium nitrite standard curve, with a lower limit of detection of 1.2 μM.

Hydrogen peroxide assay

Levels of hydrogen peroxide production in cell culture were determined as previously described (Werner 2003), with slight modifications. Briefly, cells were seeded in a 96-well plate ($0.75x10^5$ cells per well) and incubated for 24 hours at 37°C. Cells were then washed once with warm HBSS, and then 50 μL of HBSS was added to each well, followed by 50 μL of control (HBSS) or treatment. To each well, 100 μL of assay mix (200 μM homovanillic acid, 10 U/mL horseradish peroxidase, 2 mM HEPES, pH 7.5) with or without catalase (10,000 U/mL), was added. Cells were incubated for 3 hours at 37°C. Following incubation, 16 μL of stop solution (0.1 M glycine, pH 10) was added to each well, and the plates were read at 321 nm excitation, 421 nm emission. Results are calculated as catalase-inhibitable florescence and reported as a percent of control values.

Immunoblotting

Immunoblotting was performed, as previously reported (Qin et al. 2004). Samples were diluted 1:4 with 4X NuPage sample buffer and 10 μg/lane were electrophoresed on Nu-PAGE 4-12% Bis-Tris gradient gels using MOPS buffer (Invitrogen, Carlsbad, CA). Samples were transferred to nitrocellulose membranes by semi-dry transfer, blocked with 5% nonfat milk for 1 hr at 24°C, followed by incubation overnight with a primary

antibody (1:1000) at 4°C. Blots were then incubated with horseradish peroxidase-linked mouse anti-rabbit (1:5000) for 1 hr (24°C) and ECL+Plus reagents (Amersham Biosciences Inc., Piscataway, NJ) were used as a detection system.

Quantitative Real-time PCR

mRNA levels of TNFα and MIP-1α were measured by reverse transcriptase polymerase chain reaction (RT-PCR). Total RNA was extracted from the mouse olfactory bulbs using the RNA Easy kit (Qiagen, Valencia, CA) as described previously (Qin et al. 2004). The RNA was reverse transcribed from an oligo-dT primer using Omniscript (Qiagen, Valencia, CA) according to the manufacturer's instructions. Following reverse transcription, 1.5 μL of the 20 μL reaction was used with the SYBR green DNA PCR kit (Applied Biosystems, Foster City, CA) for real-time PCR analysis, per manufacturer instructions. The reaction used 0.25μM of forward and reverse primers. Cycling parameters were 1 cycle at 95ºC for 5 minutes, followed by 35 cycles of 94ºC (20 seconds), 55ºC (30 seconds), 72ºC (30 seconds), and a final extension at 72ºC for 7 minutes. RT-PCR products were analyzed by agarose gel electrophoresis (data not shown). The primer sequences used in this study were as follows: rat TNFα, 5'-TCGTAGCAAACCACCAAGCA-3' (forward) and 5'-CCCTTGAAGAGAACCTGGGAGTA-3' (reverse); rat MIP1-α, 5'-GCTTGAGCCCCAGAACATTC-3' (forward) and 5'-GATGTGGCTACTTGGCAGCAA-3' (reverse); rat β-actin, 5'-TCCTCCTGAGCGCAAGTACTCT-3' (forward) and 5'- GCTCAGTAACAGTCCGCCTAGAA-3' (reverse). The relative differences in expression between groups were expressed using cycle time (Ct) values normalized with β-actin, and relative differences between control and treatment groups were calculated and expressed as fold increases setting control as 1.

III. **SUPPLEMENTAL FIGURES**

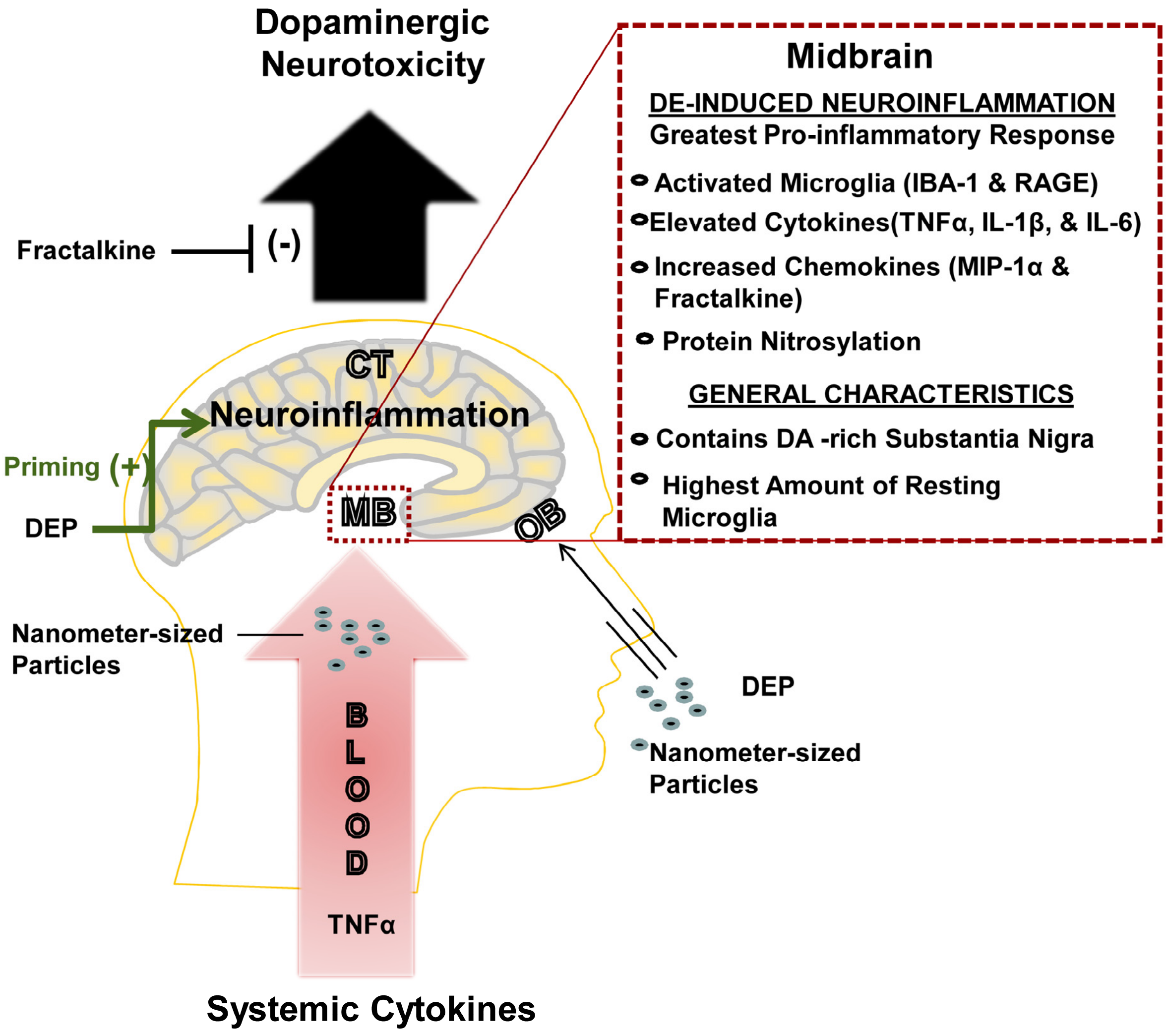

Figure S1. Diesel exhaust causes neuroinflammation through diverse pathways. Diesel Exhaust (DE), a major contributor to urban air pollution, causes microglial activation, oxidative stress (protein nitration), upregulation of pattern recognition receptors (RAGE), elevation of neurotoxic cytokines, and heightened expression of chemokines in the brain. While DE causes generalized neuroinflammation, the midbrain (containing the substantia nigra) has the highest amount of microglial markers at rest and the greatest pro-inflammatory response to DE. Nanometer-sized particles and adsorbed compounds are components of DEP predicted to reach the brain through circulating blood (and leaky blood brain barrier) and/or nasal entry through the olfactory bulb. At a cellular level, these DE components activate microglia and are toxic to DA neurons. Peripheral cytokines such as TNFα are produced by exposure to DEP, corresponding with elevation of brain TNFα. In fact, transfer of inflammation to the brain from the periphery may be amplified by particulate matter, as DEP interact with ongoing microglial activation to amplify the pro-inflammatory response (i.e. priming), which is neurotoxic. However, at the short DE exposure times tested in the current study, DA neurotoxicity is not evident. This may be due in part to the fact that DE also elevates brain fractalkine levels in the midbrain, which mitigates DEP-induced neurotoxicity. Together, these findings reveal complex, interacting mechanisms underlying air pollution-induced neuroinflammation, offering unique insight into the homeostatic mechanisms regulating DA neuron survival in the midbrain and the potential etiology of Parkinson’s disease.

III. SUPPLEMENTAL REFERENCES

Stevens T, Krantz QT, Linak WP, Hester S, Gilmour MI. 2008. Increased transcription of immune and metabolic pathways in naive and allergic mice exposed to diesel exhaust. Toxicol Sci 102(2): 359-370.

Gottipolu RR, Wallenborn JG, Karoly ED, Schladweiler MC, Ledbetter AD, Krantz T, et al. 2009. One-month diesel exhaust inhalation produces hypertensive gene expression pattern in healthy rats. Environ Health Perspect 117(1): 38-46.

Arimoto T, Kadiiska MB, Sato K, Corbett J, Mason RP. 2005. Synergistic production of lung free radicals by diesel exhaust particles and endotoxin. Am J Respir Crit Care Med 171(4): 379-387.

Block ML, Wu X, Pei Z, Li G, Wang T, Qin L, et al. 2004. Nanometer size diesel exhaust particles are selectively toxic to dopaminergic neurons: the role of microglia, phagocytosis, and NADPH oxidase. FASEB J 18(13): 1618-1620.

NIST. year. Certificate of Analysis, SRM 2975 Diesel Particulate Matter (Industrial Forklift), National Institute of Standards and Technology, Gaithersburg MD, 07 November 2000.

Liu B, Wang K, Gao HM, Mandavilli B, Wang JY, Hong JS. 2001. Molecular consequences of activated microglia in the brain: overactivation induces apoptosis. J Neurochem 77(1): 182-189.

Shapiro LA, Perez ZD, Foresti ML, Arisi GM, Ribak CE. 2009. Morphological and ultrastructural features of Iba1-immunolabeled microglial cells in the hippocampal dentate gyrus. Brain Res 1266: 29-36.

Qin L, Liu Y, Wang T, Wei SJ, Block ML, Wilson B, et al. 2004. NADPH oxidase mediates lipopolysaccharide-induced neurotoxicity and proinflammatory gene expression in activated microglia. J Biol Chem 279(2): 1415-1421.

Green LC, Wagner DA, Glogowski J, Skipper PL, Wishnok JS, Tannenbaum SR. 1982. Analysis of nitrate, nitrite, and [15N]nitrate in biological fluids. Anal Biochem 126(1): 131-138.

Werner E. 2003. Determination of cellular H2O2 production. Sci STKE 2003(168): PL3.
